# Cancer, Infant Mortality and Birth Sex-Ratio in Fallujah, Iraq 2005–2009

**DOI:** 10.3390/ijerph7072828

**Published:** 2010-07-06

**Authors:** Chris Busby, Malak Hamdan, Entesar Ariabi

**Affiliations:** 1 Department of Molecular Biosciences, University of Ulster, Cromore Rd, Coleraine, BT52 1SA, UK; 2 100 Tanfield Avenue, Neasden, London, NW2 7RT, UK; E-Mail: malakhamdan@hotmail.com; 3 82 Goldsmith Road, London, N11 3JN, UK; E-Mail: intisar_alobady@yahoo.com

**Keywords:** Fallujah, Iraq, cancer, leukemia, depleted uranium, gulf war

## Abstract

There have been anecdotal reports of increases in birth defects and cancer in Fallujah, Iraq blamed on the use of novel weapons (possibly including depleted uranium) in heavy fighting which occurred in that town between US led forces and local elements in 2004. In Jan/Feb 2010 the authors organised a team of researchers who visited 711 houses in Fallujah, Iraq and obtained responses to a questionnaire in Arabic on cancer, birth defects and infant mortality. The total population in the resulting sample was 4,843 persons with and overall response rate was better than 60%. Relative Risks for cancer were age-standardised and compared to rates in the Middle East Cancer Registry (MECC, Garbiah Egypt) for 1999 and rates in Jordan 1996–2001. Between Jan 2005 and the survey end date there were 62 cases of cancer malignancy reported (RR = 4.22; CI: 2.8, 6.6; p < 0.00000001) including 16 cases of childhood cancer 0–14 (RR = 12.6; CI: 4.9, 32; p < 0.00000001). Highest risks were found in all-leukaemia in the age groups 0–34 (20 cases RR = 38.5; CI: 19.2, 77; p < 0.00000001), all lymphoma 0–34 (8 cases, RR = 9.24;CI: 4.12, 20.8; p < 0.00000001), female breast cancer 0–44 (12 cases RR = 9.7;CI: 3.6, 25.6; p < 0.00000001) and brain tumours all ages (4 cases, RR = 7.4;CI: 2.4, 23.1; P < 0.004). Infant mortality was based on the mean birth rate over the 4 year period 2006–2009 with 1/6th added for cases reported in January and February 2010. There were 34 deaths in the age group 0–1 in this period giving a rate of 80 deaths per 1,000 births. This may be compared with a rate of 19.8 in Egypt (RR = 4.2 p < 0.00001) 17 in Jordan in 2008 and 9.7 in Kuwait in 2008. The mean birth sex-ratio in the recent 5-year cohort was anomalous. Normally the sex ratio in human populations is a constant with 1,050 boys born to 1,000 girls. This is disturbed if there is a genetic damage stress. The ratio of boys to 1,000 girls in the 0–4, 5–9, 10–14 and 15–19 age cohorts in the Fallujah sample were 860, 1,182, 1,108 and 1,010 respectively suggesting genetic damage to the 0–4 group (p < 0.01). Whilst the results seem to qualitatively support the existence of serious mutation-related health effects in Fallujah, owing to the structural problems associated with surveys of this kind, care should be exercised in interpreting the findings quantitatively.

## 1. Introduction

There have been several media reports of apparent excess rates of cancers and birth defects in the town of Fallujah in Iraq, some 50 miles west of Baghdad [1–3]. In 2004, one year after the end of the second Persian Gulf War in March 2003 there was heavy fighting between US led occupation troops and Iraqi elements in this town. Little is known about the types of weapons deployed, but reports began to emerge after 2005 of a sudden increase in cancer and leukaemia rates.

Concerns have been expressed for some time about increases in cancer, leukemia and congenital birth anomalies in Iraq. These have been blamed [4] on mutagenic and carcinogenic agents (like depleted uranium) employed in the wars of 1991 and 2003. Increases in childhood leukaemia in Basrah have recently been investigated [5] and the findings confirm that there has indeed been a significant increase since 1991. Unfortunately, since many reports from Iraq and Fallujah have been anecdotal, and have rarely been backed up by any population-based epidemiological evidence, it is difficult in these cases to assess the validity of the various assertions. Questionnaire survey studies have a long history of use in areas where there are difficulties obtaining accurate population numbers or illness rates [6]. Epidemiology in post-conflict areas where official population, cancer and birth data are not available can use questionnaire survey methods developed and used earlier in a number of areas of the UK and Ireland. The method is described fully with a sample questionnaire in Busby 2006 [7] where breast cancer rates in the town of Burnham on Sea, Somerset were reported. The study was later investigated by the official South West Cancer Intelligence Service and was shown to have given an accurate result for the breast cancer incidence rates.

For these reasons we decided to conduct such a survey study in Fallujah.

## 2. Method

### 2.1. The Survey and Questionnaire

Between Jan 20th and Feb 20th 2010 a team of 11 researchers visited houses in an area of Fallujah Iraq. They administered a questionnaire in Arabic on cancer and birth outcomes including infant mortality. It was explained that the purpose of the project was to obtain information which would show what the rates of cancer and birth effects were, that all personal information would remain completely confidential and that the results would be made available when the study was completed. The interviewer and the household member then filled out the questionnaire together. The interviewee then gave their personal identification number and the address of the house was recorded. In general people were anxious to cooperate in order to discover the true level of cancer and birth problems in the area. This has generally been found to be the case in other surveys of this type [7]. However, it was found that in some areas there was considerable distrust and fear that the questions were part of some secret-service operation and householders refused to participate; on one occasion the interview team was physically attacked. Following this, the teams were always accompanied by a local person of some reputation or standing in the community. It is estimated that the final refusal rate per house visited was less than 30%. However this 30% was almost entirely from one single area where the locals were particularly suspicious and where the teams had visited early in the survey period without a local person to vouch for the study. The final number of houses responding to the questionnaire was 711 and the total population in the resulting sample was 4,843 persons.

### 2.2. Ethical Aspects

The ethical aspects of conducting such a study were considered in some depth. In contemporary Iraq it would have been impossible to obtain ethical committee approval even if such a body existed, which it does not. The authorities have consistently avoided examining the health of communities which have complained of increases in ill health, and little has been done by the international community. Indeed, shortly after the questionnaire survey was completed, Iraqi TV reportedly broadcast that a questionnaire survey was being carried out by *terrorists* and that anyone who was answering or administering the questionnaire could be arrested. In general, the provisions of the Helsinki protocol were followed insofar as no one was coerced and all confidentiality was assured.

### 2.3. Strengths and Weaknesses

The questionnaire method has strengths and weaknesses. Its main strength is that it obtains the sex and age breakdown of the current population: in this aspect it is essentially a census of the study population at the time of the survey. No official census data would be as accurate as this, and in a post war situation no accurate census to this level of resolution exists. It also obtains the cancer data in the study population in the last ten years; the questionnaire asks for details of all cancers in the household (sex, age at diagnosis, site or type of cancer, name of clinic or doctor which diagnosed and survival).

One weaknesses of this type of study is population leakage due to migration. Although ten years is used on the questionnaire, from analysis in earlier studies of this kind [7] it has become clear that there is leakage of cases (due to deaths and subsequent population movements) and so the recent five year period is employed. However, as a consequence of such a population leakage it is clear that the result will show the *minimum* cancer rates existing in the study group. In earlier studies this effect was especially found for lung cancer which has a high mortality to incidence ratio. One other weakness is that the questionnaire could in principle be manipulated by those who do not honestly report the cancer cases in the household: no independent confirmation of the cases is made, although in principle it would be possible to do this since the individuals give their identity numbers and names of the doctors or clinics where they were treated. Our belief is that those in the present study group gave accurate answers to the questionnaire since in present day Iraq the public would be fearful of giving both misleading data and also at the same time their identities.

The population at the time of the questionnaire is used as a surrogate for the mean population over the 5-year study period and this may introduce some inaccuracies into the analysis. The question of selection bias does not arise in this case since the questionnaires were administered to a random sample of those houses in the study area selected by the interviewees and responses were random. However, the 30% who refused to respond were all from one area initially visited before it was decided to bring a local person to vouch for the study, and so it is not thought that their exclusion introduced bias. These structural problems listed above are accepted and should be borne in mind. They may be used to place limits to the accuracy of the results and we will therefore return to examine this in the Discussion Section.

### 2.4. Infant mortality

The questionnaire investigated infant mortality by asking each household if any child aged 0–1 had died in the previous ten years; again a 5-year period was employed in the analysis. The cause of death was also asked for. Researchers found that interviewees were very sensitive to the question about birth defects since there is a stigma attached to admitting to such an event in the family; a similar situation has been reported for the Hiroshima survivors [7]. There was no apparent problem with admitting an infant death, without explaining the cause, and therefore in this study, infant mortality is a better indicator of the birth outcomes than the answer to the cause of infant death and this is what was employed. For infant mortality, the mean annual birth rate was assessed from the population data as 1/5th the 0–4 population and the number of infant deaths per thousand births was obtained. This was compared with infant mortality in Egypt, Jordan and in Kuwait. Sex ratio was calculated from the population data directly.

### 2.5. Sex-Ratio

The population data in 5-year age groups was used to examine the sex ratio in 5-year birth cohorts.

### 2.6. Cancer

The national cancer rates in Iraq are not currently available; use of earlier Iraq cancer rates would bias the results since the whole country has been affected by post-war contamination following the 1991 and 2003 conflicts to various putative carcinogens, including oil fires, heavy metals and uranium from weapons. Therefore the cancer relative risks were calculated by applying 5-year sex and age group rates from the Middle East Cancer Registry (Gharbiah) [8] in Egypt for 1999 to the study group population to give an expected number of cases of all malignancies, breast cancer, leukemia, lymphoma and brain tumours in 5 years. The reported number in the previous 5-year period in the study group was then divided by this expected number to give a relative risk RR for the cancer. Standard contingency table statistical methods were employed to assess the results. The MECC Egypt age and sex specific rates were compared with the rates in Jordan [9] to check that there were no anomalous rates in Egypt which might bias the results. Age specific incidence rates for the cancers studied were broadly similar in Egypt and in Jordan [9]. The rates in Kuwait were not used since it was thought that the standard of living in Kuwait and also its proximity to contamination from the 1991 and 2003 Gulf Wars might make its use as a standardizing population questionable.

## 3. Results and Discussion

The population base obtained from the questionnaires was 711 households with 4,843 persons. The sex and age breakdown by 5-year groups is given in Table 1. Reported cancers from Jan 1st 2005 to the end of January 2010 are given in Table 2. Cancers reported before 2005 were not included. All cancer and infant death cases reported were checked against duplicate sex and age patterns to ensure there was no double reporting; if there was any doubt, data was discarded (one such instance was found). Table 3 shows the infant mortality cases reported from 2004 and includes reports of deaths in the first two months of 2010.

In Table 4 the reported numbers of cancers are compared with expected numbers for 5-year period 2005 to the sample cutoff date in 2010. The expected numbers are calculated by applying the sex and 5-year age group rates obtained from the Middle East Cancer Consortium [8] for Egypt 1999 and also checked against rates in Jordan [9] 1996–2001.

Table 5 shows the mean infant mortality rate per 1000 births for the period 2006–2010 including deaths reported in the first two months of 2010. Also shown are rates for the period from 1st January 2009 and comparisons are made with infant mortality rates in Jordan, Egypt and Kuwait.

The responses show that there is an anomalous sex ratio in the 0–4 age group. There are 860 males to 1000 females, a significant 18% reduction in the male births from the normal expected value of 1,055 (267 boys expected, 234 observed; p < 0.01) Perturbation of the sex ratio is a well known consequence of exposure of mutagenic stress and results from the sensitivity of the male sex chromosome complement to damage (the females have two X chromosomes whereas the males have only one). A number have studies have examined sex-ratio and radiation exposure of mothers and fathers. Of relevance is the study of Muller *et al.* [10] of the offspring of 716 exposed fathers who were Uranium miners. There was a significant reduction in the birth sex ratio (fewer boys). Lejeune *et al.* (1960) [11,12] examined the offspring of fathers who had been treated with pelvic irradiation; at high doses there was an increase in the sex-ratio, but this reversed in the low doses (around 200 mSv). Schull *et al.* 1966 [13] found a reduction in the sex ratio in A-Bomb survivor fathers (mothers “unexposed”) for children born 1956–1962 a reversal of an earlier finding by Schull and Neel 1958 [14] of a positive effect in the 1948–1955 births. It should be noted that there were external and internal irradiation effects in these groups, with the internal effects predominating in the later years. Yoshimoto *et al.* 1991 [15] found an overall reduction in the sex ratio for A-Bomb survivors for children born 1946–1984. Thus the evidence suggests that exposure to ionising radiation at low doses and specifically exposure to Uranium may cause a reduction in the sex ratio.

It is clear that the 0–4 population, born in 2004–2008, after the fighting, is significantly 30% smaller than the 5–9, 10–14 and 15–19 populations. This could be a result of lower fertility or early foetal losses in this cohort. It has been pointed out by a referee that it might also in principle be a result of the deaths of men in the 2004 fighting but this does not seem to be supported by the sex ratios in the men and women aged 25 and over. The infant mortality numbers reported by year point to sudden increase in deaths in 2006 (Table 3). There was only one death reported for the two years 2004 and 2005 in the sample population. For the period from 2006 to the end of the survey there was a mean death rate of 80 per 1,000 births, more than 4 times the rate in Egypt and in Jordan (p < 0.00001) and some 9 times the rate in Kuwait. The rate seems to have increases markedly after 2009 to a rate of 136 per 1,000 births. These results support the many reports of congenital illness and birth defects in Fallujah and suggest that there is evidence of genetic stress which appeared around 2004, one year before the effects began to show.

The results for cancer show some alarming rates in the 5-year period. Relative Risk based on the Egypt and Jordan cancer rates are significantly higher for all malignancy, leukaemia, lymphoma, brain tumours and female breast cancer. Between January 2005 and the survey end date there were 62 cases of cancer (all malignancies) reported (RR = 4.22; CI: 2.8, 6.6; p < 0.00000001) including 16 cases of childhood cancer 0–14 (RR = 12.6; CI: 4.9, 32; p < 0.00000001). Highest risks were found in all leukaemias in the age groups 0–34 (20 cases RR = 38.5; CI: 19.2, 77; p < 0.00000001), all lymphomas 0–34 (8 cases, RR = 9.24;CI: 4.12, 20.8; p < 0.00000001), female breast cancer 0–44 (12 cases RR = 9.7;CI: 3.6, 25.6; p < 0.00000001) and brain tumours all ages (4 cases, RR = 7.4;CI: 2.4, 23.1; P < 0.004). These results for cancer also support the idea that there has been exposure to some mutagenic agent at some time in the past. Could this have been around 2004 when the fighting occurred? The answer depends upon whether it is plausible to accept such a short time lag between exposure and clinical expression of the cancer, leukaemia or lymphoma. It is commonly believed that the lag between initiation and expression of cancer is a significant period: for exposure to acute external low LET radiation the onset of leukaemia is stated to be about 5 to 7 years, and for breast cancer and solid tumours as high as 15 to 20 years. However, genetic damage expansion models for cancer [16,17] hold that it is the acquisition of a key number of mutations which lead to final clinical expression. This is then seen as purely probabilistic so long as the mutagenic stresses are constant; in this way the exponential increases in cancer rates with age are explained as are cancer rates and initiation expression lags in cell populations with different natural replication rates. However, such an explanation makes it also clear that the sudden (spike) introduction of a mutagenic stress could supply a final key mutation in those individuals who already carry almost the full necessary complement of mutations for the specific cancer [18]. This idea explains many observations of increases in cancer shortly (a few years) after an exposure. For example, there seems to have been a rapid increase in lymphoma in Italian peacekeepers potentially exposed to depleted uranium in the Balkans [19]. Tondel *et al.* have reported increased cancer risk in Northern Sweden peaking less than 5 years after the Chernobyl contamination and significantly associated with the levels of Caesium-137 fallout in municipalities [20]. Despite the assertions of the studies of the Japanese A-Bombs (which did not begin until 1952) that the first increases in leukaemia in the study group appeared more than 5 years after the bomb, leukemia in victims of Hiroshima and Nagasaki was reported beginning only months after the explosion, and even in those who had not been exposed to the prompt radiation but to fallout and uranium in the bombed city debris [21]. Furthermore, the onset lag for internal exposure to high LET radiation (e.g., Uranium) has not been determined and it can be argued that this lag cannot be deduced from the external low LET studies that make up the current radiation risk model. On the other hand, it may be that the increases in cancer found here for some individuals are the result of some earlier exposure, perhaps during the 1991 Gulf War. The origin and time of introduction of the carcinogenic agent causing the effects found here will be the subject of a separate report. However it does not seem unreasonable to conclude that the causes of the infant deaths and the cancer increases are one and the same.

We must finally address the earlier listed shortcomings of the interview questionnaire survey method. These might have been of concern had the findings been less clear but since the Relative Risks for the various indicators were extremely high, it can hardly be possible that these results could have occurred through errors introduced through any of the potential problems outlined in the Methods Section. A 100% error in the population would only halve the relative risks. The levels of cancer and infant mortality which have been found are too great to be accommodated by any hypothesis except that a significant proportion of those interviewed completely invented the results, and for the reasons already given *i.e.*, that they had given names, addresses and identities and the names of the doctors and clinics involved in an area where the consequences of giving misleading responses to questions are severe, this seems highly unlikely.

## 4. Conclusions

This study was intended to investigate the accuracy of the various reports which have been emerging from Fallujah regarding perceived increases in birth defects, infant deaths and cancer in the population and to examine samples from the area for the presence of mutagenic substances that may explain any results. We conclude that the results confirm the reported increases in cancer and infant mortality which are alarmingly high. The remarkable reduction in the sex ratio in the cohort born one year after the fighting in 2004 identifies that year as the time of the environmental contamination. In our opinion, the magnitude of these effects make it difficult to question them on the basis of any of the hypothetical shortcomings of the study type which we have considered although these must be borne in mind. However, owing to the various constraints placed by circumstance on the methods employed, we must emphasise that the results of this study should be interpreted with those aspects in mind. Finally, the results reported here do not throw any light upon the identity of the agent(s) causing the increased levels of illness and although we have drawn attention to the use of depleted uranium as one potential relevant exposure, there may be other possibilities and we see the current study as investigating the anecdotal evidence of increases in cancer and infant mortality in Fallujah.

## Figures and Tables

**Table 1:** Sex and 5-year age group population in the Fallujah response sample; also calculated is the Sex ratio (males per 1000 females) in the four groups aged 0–19.

**Age Group**	**Males**	**Females**	**Sex Ratio**
0–4	234	272	860
5–9	481	407	1,182
10–14	388	350	1,109
15–19	393	389	1,010
20–24	166	213	
25–29	182	224	
30–34	129	106	
35–39	157	93	
40–44	71	133	
45–49	144	67	
50–54	61	58	
55–59	31	13	
60–64	31	10	
65–69	9	6	
70–74	17	0	
75–79	3	1	
80–84	1	2	
85+	1	0	
Total	2,499	2,344	

**Table 2:** Cancers reported in responses from January 1st 2005 to January 31st 2010.

**Cancer**	**Males**	**Females**	**Total**
All malignancy all ages	28	34	62
Childhood cancer ages 0–14	6	10	16
Leukemias all ages	16	6	22
Lymphomas all ages	9	1	10
Brain tumours all ages	2	2	4
Breast cancer (f) all ages	0	13	13

**Table 3:** Infant deaths reported from 2004.

**Year reported died (Approximate birth year)**	**Number of infant deaths 0–1 years**
2004 (2003)	1
2005 (2004)	0
2006 (2005)	8
2007 (2006)	4
2008 (2007)	6
2009 (2008)	10
2010 first 2 months only (2009)	6

**Table 4:** Relative Risks of cancer in Fallujah 2005–2010. Reported (Rep) and expected (Exp) numbers of cases and statistics for main classes of cancer and leukaemia/lymphoma observed. Expected numbers calculated on the basis of rates for 1999 in Egypt and checked against rates reported for Jordan 1996–2001.

**Cancer**	**Rep**	**Exp**	**RR**	**95% CI**	**Chisq**	**p-value <**
All malignancy all ages	62	14.7	4.2	2.8 < RR < 6.6	50.9	0.00000001
Childhood cancer 0–14	16	1.27	12.6	4.9 < RR < 32	46.3	0.00000001
Breast cancer (f) all ages	13	2.46	5.3	2.4 < RR < 11.8	20.75	0.00002
Breast cancer (f) 0–44	12	1.24	9.7	3.6 < RR < 25.6	30.9	0.00000002
Leukaemia all ages	22	0.99	22.2	12.1 < RR < 41	212	0.00000000
Leukaemia 0–35	20	0.52	38.5	19.2 < RR < 77	287	0.00000000
*Lymphoma all ages	9	2.11	4.27	1.3 < RR < 14	6.95	0.008
*Lymphoma 0–35	8	0.865	9.24	4.12 < RR < 20.8	43.8	0.00000000
Brain tumours all ages	4	0.542	7.4	2.4 < RR < 23.2	16.2	0.004

* The class Lymphoma may be contaminated with lymphatic metastases of common tumours.

**Table 5:** Infant deaths and births 1st January 2006 to 28th February 2010 with comparisons with Egypt, Jordan and Kuwait. Mean annual birth rate is calculated from the reported 0–4 population.

**Birth and deaths information**	**Value**
0–4 population reported	506
Mean Annual birth rate	101.2
Births in the period 2006–2010+ (50 months)	425
Reported deaths in the period	34
Rate per thousand births in Fallujah 2006–2010+	80
Reported deaths in the period 2009–2010+ (14 months)	16
Rate per thousand births in Fallujah 2009–2010+ (14 months)	136
Rate in Kuwait 2008	9.7
Rate in Egypt 2008	19.8
Rate in Jordan 2008	17
